# Metabolic Syndrome Prevalence and Cardiovascular Risk Assessment in HIV-Positive Men with and without Antiretroviral Therapy

**DOI:** 10.3390/medicina57060578

**Published:** 2021-06-05

**Authors:** Win-Long Lu, Yuan-Ti Lee, Gwo-Tarng Sheu

**Affiliations:** 1 Institute of Medicine, Chung Shan Medical University, No. 110, Section 1, Jianguo N. Road, Taichung City 402, Taiwan; winlonglu@gmail.com; 2 School of Medicine, Chung Shan Medical University, No. 110, Section 1, Jianguo N. Road, Taichung City 402, Taiwan; 3 Division of Infectious Diseases, Department of Internal Medicine, Chung Shan Medical University Hospital, No. 110, Section 1, Jianguo N. Road, Taichung City 402, Taiwan; 4 Department of Medical Oncology and Chest Medicine, Chung Shan Medical University Hospital, No. 110, Section 1, Jianguo N. Road, Taichung City 402, Taiwan

**Keywords:** HIV, cardiovascular disease, metabolic syndrome, HAART, Framingham risk score

## Abstract

Treatment of HIV infection is a lifelong process and associated with chronic diseases. We evaluated the prevalence and predictors of metabolic syndrome (MetS) and cardiovascular diseases (CVDs) with individual antiretroviral drugs exposure among HIV-infected men in Taiwan. A total of 200 patients’ data were collected with a mean age of 32.9. Among them, those who had CD4 positive cell number less than 350/mL were eligible to have highly active antiretroviral therapy (HAART). Patients were divided into group-1 that contains 45 treatment-naïve participants, and group-2 that includes 155 HAART treatment-experienced participants. MetS prevalence between group-1 and group-2 was 18% and 31%, respectively. The Framingham Risk Score (FRS) for the naïve and experienced groups were 4.7 ± 4.2 and 3.87 ± 5.92, respectively. High triglyceride (TG > 150 mg/dL) in group-1 and group-2 were 15.6% and 36.6% (*p* < 0.05), whereas, lower high-density lipoprotein (HDL < 39 mg/dL) in group-1 and group-2 presented as 76.7% versus 51% (*p* < 0.05), respectively. In group-2, treatment with protease inhibitors (PIs) resulted in higher TG levels when compared with non-nucleotide reverse transcriptase inhibitors (NNRTIs) and integrase inhibitors (InSTIs). The prevalence of MetS in the treatment-naïve group was lower than that of the treatment-experienced group; high TG level resulted in higher MetS prevalence in the treatment-experienced group. In contrast, the cardiovascular risk of FRS in the treatment-naïve group was higher than that of the treatment-experienced group, which may result from the low HDL level. Although group-1 participants have a higher risk of developing CVDs, in group-2, an increasing TG level in PIs user indicated higher CVDs risk. TG and HDL are two significant biofactors that required regular evaluation in HIV-positive individuals.

## 1. Introduction

According to the WORLD AIDS DAY 2020 fact sheet, in 2019, 38.0 million [31.6–44.5 million] people globally were living with human immunodeficiency virus (HIV). A total of 75.7 million [55.9–100 million] people have become infected with HIV since the start of the epidemic. HIV infection is a lethal disease, and approximately 32.7 million people have died from acquired immune deficiency syndrome (AIDS)-related illnesses since the start of the epidemic [1]. Fortunately, combined antiretroviral therapy (ART) improves health, prolongs life and substantially reduces the risk of HIV transmission with spectacular success [2]. The survival time of the HIV/AIDS individuals became longer by 10 years in the past 20 odd years [3]. People nowadays believe the end of AIDS is possible [4]. However, a new set of HIV-associated complications have emerged, such as cardiomyopathy, heart failure [5,6,7,8] and diabetes mellitus [9,10,11,12] resulting in a chronic disease that spans several decades of life, and has become an important health issue.

Among HIV-infected individuals, the mortality due to cardiovascular diseases (CVDs) ranges between 6% and 15%, and the incidence of CVD is higher than that among noninfected individuals; moreover, HIV-infected individuals are usually affected at a younger age [13,14]. Irrespective of HIV-1 status, infants with vertically transmitted HIV have significantly worse cardiac function than other infants have recorded [15]. A retrospective cohort study of the HIV-infected patients residing in the Local Health Authority of Brescia, northern Italy, from 2000 to 2012, reported that overall cardiovascular event (CVE) risk in HIV-positive patients was twice as high as CVE risk in the general population [16].

With the application of effective highly active antiretroviral therapy (HAART), people living with HIV are ageing, but the most frequent complications are metabolic abnormalities related to the chronicity of the infection, including metabolic syndrome (MetS) [17,18,19], lipodystrophy [20] and mitochondrial toxicity [21,22,23].

The U.S. National Cholesterol Education Program Adult Treatment Panel III (ATPIII) report identified MetS as a multifaceted risk factor for CVDs [24]. An international cross-sectional study that used a well-characterized cohort of 788 HIV-infected adults recruited at 32 centers reported that the prevalence of MetS was 14% according to the International Diabetes Federation (IDF) criteria and 18% by the U.S. ATPIII criteria [25]. In addition, the prevalence of MetS among those HIV-infected patients (17%) was similar to that previously reported in uninfected individuals [26]. Furthermore, the overall prevalence of MetS was reported to be similar among an HIV-infected group and HIV-seronegative group (25.5% vs. 26.5%, respectively) from the US outpatient population [27]. Although high prevalence of CVDs is associated with HIV infection, the above-mentioned reports found no significant differences in MetS prevalence among HIV-infected and noninfected groups.

Interestingly, a higher prevalence of MetS was observed in the HAART group (ATPIII—33.3%; IDF—36.4%) than in the HAART-naïve group (ATPIII—2%; IDF—12%) in a cross-sectional study carried out in a teaching hospital in North India [28] Furthermore, another study found that the prevalence of MetS among HAART-exposed patients was 19.3%, while it was 5.3% among HAART naïve patients, within African populations with a majority of female patients (64%) [29]. The above reports indicated that HAART is associated with a higher prevalence of MetS. Therefore, analyzing the prevalence of MetS among HIV-infected patients from different ethnic populations and HAART patients is still an important issue.

HIV infection itself and HAART can both modify the risk of CVDs [14]. HAART regimes inhibit viral replication by acting at different stages with different combinations of drugs, as reported previously [2,30]. HAART regimes have been classified in different therapeutic groups according to their mechanisms of action: nucleoside reverse transcriptase inhibitors (NRTIs), nonnucleoside reverse transcriptase inhibitors (NNRTIs), protease inhibitors (PIs), fusion inhibitors, entry inhibitors and integrase strand transfer inhibitors (InSTIs) [30]. The role of HAART in promoting atherosclerosis and CVDs has been extensively reviewed [31]; HAART is associated with increase of total cholesterol (CHO), low-density lipoprotein (LDL) and triglycerides (TG) and reduced values of high-density lipoprotein (HDL) [32]. Therefore, the present study focuses on MetS prevalence, CVD risk and characteristics of the possible related factors, including individual antiretroviral drug exposure in HIV-infected men.

## 2. Materials and Methods

### 2.1. Study Design

This was a prospective cross-sectional study of metabolic syndrome and cardiovascular disease risk factors in HIV-positive men attending a tertiary care hospital in central Taiwan. The study protocol was reviewed and approved by the Hospital’s Research and Ethics Committee (IRB approval number CS14034).

### 2.2. Study Population

The study population was made up of male adult patients and was diagnosed as HIV-1 positive by Western blot or polymerase chain reaction analysis at the hospital.

### 2.3. Data Collection

In this study, a survey was used to collect 200 copies of case reports from the HIV-infected patients between 2014 and 2016. The research analyzed the compliance of medication, metabolic syndrome, cardiovascular disease, and treatment of viral resistance through systematic follow-up of medical guidance cases. The inclusion criteria were: (1) age greater than or equal to 20 years old; (2) diagnosis of AIDS confirmed (ICD9 042); (3) only cases in this hospital and patients who have been treated for more than 6 months; (4) cases of HIV-infected patients taking either HAART (Experienced) or not taking cocktail therapy (naïve) who are willing to accept the investigation and service. The exclusion criteria were: (1) male patient younger than 20 years old; (2) when the subject, legal representative or person with consent is unable to read; (3) incomplete data, or unable to assess efficacy; (4) patient is tracked for less than 6 months in the hospital. This is a noninvasive treatment plan, so no withdrawal or rescue treatment conditions were included. Questionnaires were introduced to the subjects to obtain basic demographic data and history of education, occupation, HAART type and duration, cigarette smoking, alcohol drinking, exercise, antihypertensive and diabetic medication. Thereafter, blood pressure was taken in the sitting position after five minutes of rest. Weight, height and waist circumference were measured to calculate body mass index (BMI). MetS was defined as the presence of 3 or more of the following 5 abnormalities for men: (1) waist ≥ 90 cm, (2) systolic blood pressure (SBP) ≥ 131 mmHg or diastolic blood pressure (DBP) ≥ 81 mmHg, (3) HDL < 40 mg/dL, (4) fasting glucose ≥ 100 mg/dL, and (5) triglyceride (TG) ≥ 150 mg/dL. The HAART backbone is NRTIs (Zidovudine; Lamivudine; Abacavir; Tenofovir/Emtricitabine) that was combined with PIs (Atazanavir; Lopinavir/Ritonavir; Darunavir) or NNRTIs (Nevirapine; Efavirenz; Rilpivirine; Efavirenz/Tenofovir/Emtricitabine) or InSTIs (Raltegravir).

### 2.4. Statistical Analysis

Data from the completed questionnaires and laboratory results were categorized as HIV-positive treatment-naïve (group-1) and HIV-positive treatment-experienced (group-2). Ten-year risk assessments for CVD was performed by the Framingham risk score (FRS) calculator [33] using age, diabetes, smoking, SBP, CHO, LDL, and HDL as predictors. Statistical analyses were performed using SPSS version 18 (Chicago, IL, USA). Continuous variables were compared using the Mann–Whitney U test for non-normally distributed variables. The chi-squared test was used to determine whether there is a significant difference between one or more categories with numbers indicated. The level of statistical significance was established at the *p* value of <0.05. One-way ANOVA was used to compare the mean values between the three subgroups and calculated by the Tukey honest significant difference test (HSD).

## 3. Results

### 3.1. Epidemiology of Participants

During the period from June 2014 to April 2016, a total of 200 HIV infected men in the hospital signed the consent form. Before 2016, patients of Taiwan who had CD4 positive cell number less than 350/mL were eligible to have HAART therapy. After evaluating their anti-HIV therapy, patients were divided into group-1 (*n* = 45) those not taking cocktail therapy (Naïve), and group-2 (*n* = 155) those taking HAART (Experienced), respectively (Table 1). After 2015, the HAART was applied to every HIV positive patient without restriction of CD4 positive cell number. The overall mean (±SD) age was 32.9 (±8.2) years; the group-1 mean (±SD) age was 30.5 (±7.6) years old and the group-2 mean (±SD) age was 33.6 (±8.2) years old; group-1 was significantly younger than group-2. Our data showed that the mean of HAART treatment period was 3.3 y (±2.1) that may reflect why group-1 was younger than group-2. Among them, only 10 individuals were married. A total of 66% of participants had received college education and the student status in group-1 (17.8%) was significantly higher than that in group-2 (7.1%). A total of 77.5% participants had a full-time job, 51% were smokers and 41% of participants drank alcohol. We found no significant difference in regular excise habits between the two groups.

### 3.2. Basic Physiological Data of the Participants

The overall mean (±SD) waist circumference was 80.9 cm (±6.1), height was 171.8 cm (±6.1) and weight was 67.5 kg (±12.6) for all participants (Table 2). The calculated mean BMI for all participants was 22.8 (±3.8), with no significant difference in BMI in group-1 (23.1 ± 4.3) and group-2 (22.7 ± 3.7). There were also no significant differences in average SBP, DBP and heartbeat in the two groups.

### 3.3. Laboratory Variables of the Participants

The TG analysis data were 92 (median) and 115 mg/dL in group-1 and group-2, respectively. In group-1, 15.6% of participants had a high TG level (≧151 mg/dL), whereas, in group-2, 36.6% of patients presented a high level, resulting in a significant difference (Table 3). Although no significant differences were found in CHO, LDL and fasting blood glucose (glucose), there was a significant difference in the HDL levels between the two groups. The HDL level in group-1 was 34.3 mg/dL (median) and it was 39.8 mg/dL in group-2 (*p* < 0.05). The median number of CD4+ cells in both groups showed no significant difference, but the median plasma HIV-RNA viral load (VL) was significantly different, with 20,535 copies/mL in group-1 and 20 copies/mL in group-2. In group-2, 63.2% of patients have less than 20 copies/mL of VL, 20.6% had 21–1000 copies/mL of VL and 16.1% had more than 1000 copies/mL of VL. Two participants lacked TG and CHO data and four participants had no HDL, LDL and glucose data.

### 3.4. Prevalence of Metabolic Syndrome in HIV-Positive Patients

The numbers of all participants with MetS were analyzed (Table 4). In total, 56 individuals (28%) had MetS and the positive percentage was correlated with older age. When the MetS was analyzed in group-1 and group-2 (Table 4), 18% of participants in group-1 had MetS and the prevalence was 31% in group-2. The correlation of aging and MetS in naïve and HAART participants was further analyzed as listed in Table 4 with *p* value. HAART significantly increased metabolic syndrome prevalence in men over 50 years of age.

### 3.5. Association of Cardiovascular Risk in HIV Therapy

Sufficient data were available for 42 participants in group-1 and 154 participants in group-2 to evaluate cardiovascular risk by FRS (Table 5). The percentage of 10-year estimated coronary heart disease (CHD) risk among all of the HIV-infected participants was 4.53. Percentage of cardiovascular risk by group was 4.70 and 3.87 for group-1 and group-2, respectively. The calculated heart age/vascular age was 38 years old, which was higher than average age (32.9 yr.) of all of the HIV-infected participants. For ages 20–30, FRS results were 1.64 and 1.43 for group-1 and group-2, respectively. For ages 31–40, FRS results were 5.32 and 4.18 for group-1 and group-2, respectively (Supplemental Table S1). The results indicate that group-1 participants without HAART treatment have a higher risk of developing CVDs. Although, only “Age” was significantly associated with cardiovascular risk but not FRS; the possible explanation is that only HDL significantly elevated by HAART, whereas other FRS factors of smoking, BMI, sugar, and cholesterol were not significantly different between these two groups as data listed in Table 1, Table 2 and Table 3.

### 3.6. Analysis of the Lipid Profile Association with HAART Drugs

Since TG level was significantly higher in group-2 who had received HAART (Table 3), the data of TG, CHO, HDL and LDL were collected from 153 HAART-treated participants (Supplemental Table S2). Sixty-two participants were treated with PIs, 72 participants were treated with NNRTIs, and 19 participants were treated with InSTIs. The TG (mean) level was 162.95 mg/dL for users of PIs, 121.75 mg/dL for users of NNRTIs and 110.32 mg/dL for users of InSTIs. An ANOVA test was conducted to test the significance of TG level among groups as listed in Supplemental Table S3. To further analyze the statistical significance for pairwise comparison of multiple treatments, post-hoc Tukey HSD method was applied, and the results are listed in Table 6. The TG was significantly higher among users of PIs than among users of NNRTIs (*p*=0.010); whereas TG was significantly lower among NNRTIs users than among users of PIs (*p* = 0.010) according to Tukey HSD calculation (Table 6). Analyses of CHO, HDL and LDL showed no statistical differences from calculation.

## 4. Discussion

In this study, we evaluated the prevalence of MetS and analyzed the associated factors among HIV-infected young men (*n* = 200) who are ethnic Chinese. The overall MetS prevalence of HIV-infected men was 28% (Table 4). We also found that 31% (48/155) of HIV-infected Taiwanese men in the HAART group had MetS; the MetS prevalence in the naïve group was 18% (Table 4). Our data were similar to those of recently reported study from North India that collected a total of 116 HIV positive male and female patients in contrast to our study group of male only patients; the prevalence of MetS was also higher in the HAART group than in the naïve group [28]. Furthermore, a previous report estimated the prevalence of MetS in Taiwan and found the age-standardized prevalence of MetS was 15.7% according to modified ATP III criteria from a nationwide cross-sectional population-based survey [34]. The prevalence of MetS in the general population was similar to that in the naïve group. Although, the group-1 (treatment-naïve) participants were significantly younger than the group-2 participants (Table 1) that might be due to higher CD4-positve count with recent HIV infection. Therefore, we further analyzed the same range of ages to reduce the bias and found higher MetS prevalence was observed in the group-2 participants (Table 4). Although aging is a factor tightly associated with metabolic syndrome in general population, our data suggest that HAART significantly increased metabolic syndrome prevalence in men over 50 years of age. According to both results, we can conclude that HAART is associated with a higher prevalence of MetS in HIV-positive Asian patients.

Other significant differences were also detected in our two groups of participants. The viral loads in group-2 were significantly lower than those of the group-1 HAART-naïve participants (Table 3). Apparently, HAART effectively reduced HIV replication. Furthermore, significantly higher TG and HDL levels were observed in group-2 (Table 3). No significant differences in smoking, SBP, CHO and blood glucose levels were found in both groups (Table 2 and Table 3), which are factors analyzed by FRS. Therefore, when CVD risk was analyzed by FRS, it was found that group-2 had lower CVD risk than group-1 (Table 5); this could be because HDL is one of the predictors but TG is not. As MetS is positively associated with CVDs, therefore, it should be noted that FRS modified with TG but not CHO may be more appropriate to evaluate CVD risk among HIV-positive patients.

HAART-associated dyslipidemia is characterized by hypertriglyceridemia, hypercholesterolemia, and decreased serum levels of HDL, either accompanied or not accompanied by increased levels of LDL [14,21]. Although our data show that only hypertriglyceridemia was observed in HAART-treated group-2, statistical comparison by Tukey HSD (Table 6) showed TG levels were higher among users of PIs than among users of NNRTIs and InSTIs. Although PIs is highly associated with hypertriglyceridemia, more drug-resistance mutations in the viral protease gene are required for PIs-resistance to develop that make PIs therapy in demand [35]. Since HIV-positive patients usually need to take HAART regularly and even for their whole lives [2], personalized medicine would provide better health management for HIV-positive patients.

This study has several limitations. First, the prospective cross-sectional study design was still composed of several uncontrolled factors, which may affect our results. For instance, the study has missing data such as glycohemoglobin (HbA1c). The HbA1c is important information for long-term blood sugar control among HIV-infected patients [12], but the aim of the study was to evaluate the prevalence and predictors of MetS and CVDs. Second, the 2015 WHO guideline has recommended antiretroviral therapy should be initiated in everyone who living with HIV at any CD4 cell count. However, this study was conducted between 2014 and 2016. Therefore, we had the data for comparing the difference between treatment-naïve and treatment-experienced HIV-positive patients.

## 5. Conclusions

The prevalence of MetS in the treatment-naïve group was lower than that of the treatment-experienced group. In contrast, the cardiovascular risk in the naïve group was higher than that of the experienced group, which may result from the low HDL level. In the treatment-experienced group, an increasing triglyceride level of PIs indicated higher CVDs risk when compared with NNRTIs and InSTIs. We suggest that FRS modified with TG but not CHO may be more appropriate to evaluate CVD risk among HIV-positive patients.

## Figures and Tables

**Table 1:** Characteristics of HIV-infected patients (*n* = 200).

Demographics		Total *n* = 200	Group-1 *n* = 45	Group-2 *n* = 155	*p* Value
Gender	Male	200 (100%)	45 (22.5%)	155 (77.5%)	
Age (yr) ± SD		32.9 ± 8.2	30.5 ± 7.6	33.6 ± 8.2	0.024 *
	20–30	81 (40.5%)	24 (53.3%)	57 (36.8%)	0.134
	31–40	94 (47.0%)	17 (37.8%)	77 (49.7%)	
	≧41	25 (12.5%)	4 (8.9%)	21 (13.5%)	
Marital status	No	190 (95.0%)	44 (97.8%)	146 (94.2%)	0.331
	Yes	10 (5.0%)	1 (2.2%)	9 (5.8%)	
Education	High school	68 (34.0%)	15 (33.3%)	53 (34.2%)	0.915
	College	132 (66.0%)	30 (66.7%)	102 (65.8%)	
Occupation	Full-time	155 (77.5%)	32 (71.1%)	123 (79.4%)	0.379
	Part-time	21 (10.5%)	5 (11.1%)	16 (10.3%)	
	Jobless	24 (12.0%)	8 (17.8%)	16 (10.3%)	
Student	No	181 (90.5%)	37 (82.2%)	144 (92.9%)	0.031 *
	Yes	19 (9.5%)	8 (17.8%)	11 (7.1%)	
Smoking	No	102 (51.0)	20 (44.4)	82 (52.9)	0.159
	Quit	23 (11.5)	3 (6.7)	20 (12.9)	
	Yes	75 (37.5)	22 (48.9)	53 (34.2)	
Drinking	No	82 (41.0)	15 (33.3)	67 (43.2)	0.421
	Quit	32 (16.0)	7 (15.6)	25 (16.1)	
	Yes	86 (42.0)	23 (51.1)	63 (40.6)	
Regular exercise	No	108 (54.0)	24 (53.3)	84 (54.2)	0.919
	Yes	92 (46.0)	21 (46.7)	71 (45.8)	

SD, standard deviation. * Statistically significant, *p* value of <0.05. Group-1: naïve; group-2: HAART-treated.

**Table 2:** Basic physiological data of the participants (*n* = 200).

Variables		Total *n* = 200	Group-1 *n* = 45	Group-2 *n* = 155	*p* Value
Mean waist circumference	(cm)	80.9 ± 6.1	80.3 ± 10.2	81.1 ± 10.0	0.635
Mean height	(cm)	171.8 ± 6.1	172.3 ± 4.7	171.6 ± 6.5	0.427
Mean weight	(kg)	67.5 ± 12.6	68.7 ± 13.0	67.2 ± 12.5	0.502
BMI	≤17	12 (6.0%)	3 (6.7%)	9 (5.8%)	0.903
	18–24	125 (62.5%)	29 (64.4%)	96 (61.9%)	
	≧25	63 (31.5%)	13 (28.9%)	50 (32.3%)	
Mean BMI		22.8 ± 3.8	23.1 ± 4.3	22.7 ± 3.7	0.539
Systolic blood pressure	≤130 mmHg	146 (73.0%)	31 (68.9%)	115 (74.2%)	0.480
	≧131 mmHg	54 (27.0%)	14 (31.1%)	40 (25.8%)	
Mean SBP	(mmHg)	122.4 ± 17.8	122.4 ± 14.3	122.3 ± 13.6	0.980
Diastolic blood pressure	≤80 mmHg	122 (61.0%)	25 (55.6%)	97 (62.6%)	0.395
	≧81 mmHg	78 (39.0%)	20 (44.4%)	58 (37.4%)	
Mean DBP	(mmHg)	78.7 ± 10.0	79.0 ± 10.7	78.6 ± 9.8	0.839
Mean heartbeat	(beat/min)	82.5 ± 12.2	84.8 ± 12.0	81.8 ± 12.3	0.148

BMI, body mass index. DBP, diastolic blood pressure. SBP, systolic blood pressure. Group-1: naïve; group-2: HAART-treated.

**Table 3:** Laboratory variables of the participants (*n* = 200).

Variables		Total	Group-1	Group-2	*p* Value
TG median	mg/dL 95% C.I.	108.5 (69.8, 165.3)	92.0 (67.0, 132.5)	115.0 (70.0, 181.0)	0.078
TG level (*n* = 198)	≤150 mg/dL	135 (68.2%)	38 (84.4%)	97 (63.4%)	0.008 *
	≧151 mg/dL	63 (31.8%)	7 (15.6%)	56 (36.6%)	
CHO median	mg/dL 95% C.I.	164.0 (140. 8185.0)	167.0 (143.5, 186.0)	164.0 (140.0, 184.0)	0.892
CHO level (*n* = 198)	≤200 mg/dL	169 (85.4%)	39 (86.7%)	130 (85.0%)	0.777
	≧201 mg/dL	29 (14.6%)	6 (13.3%)	23 (15.0%)	
HDL median	mg/dL 95% C.I.	38.4 (31.8, 45.2)	34.3 (28.6, 39.8)	39.8 (32.5, 47.1)	0.005 *
HDL level (*n* = 196)	<39 mg/dL	111 (56.6%)	33 (76.7%)	78 (51.0%)	0.003 *
	≧40 mg/dL	85 (43.4%)	10 (23.3%)	75 (49.0%)	
LDL median	mg/dL 95% C.I.	96.0 (79.3, 116.0)	101.0 (78.0, 127.0)	93.0 (79.5, 114.0)	0.074
LDL level (*n* = 196)	≤100 mg/dL	114 (58.2%)	20 (46.5%)	94 (61.4%)	0.080
	≧101 mg/dL	82 (41.8%)	23 (53.5%)	59 (38.6%)	
Glucose median (*n* = 196)	mg/dL 95% C.I.	98 (73,325)	97 (77,135)	99 (73,325)	0.471
CD4+ median	Cells/mm^3^ 95% C.I.	472.5 (342.0, 633.8)	442.0 (338.5, 601.0)	479.0 (351.0, 642.0)	0.391
CD4+ level	<200 cells/mm^3^	15 (7.5%)	2 (4.4%)	13 (8.4%)	
	200–500 cells/mm^3^	96 (48.0%)	26 (57.8%)	70 (45.2%)	0.291
	>500 cells/mm^3^	89 (44.5%)	17 (37.8%)	72 (46.5%)	
VL median	Copies/mL	22.0	20,535.0	20	0.000 *
	95% C.I.	(20. 9156)	(7813, 50,567)	(20, 96)	0.000 *
VL level	≤20 copies/mL	98 (49.0%)	0	98 (63.2%)	
	21–1000 copies/mL	36 (18.0%)	4 (8.9%)	32 (20.6%)	
	>1000 copies/mL	66 (33.0%)	41 (91.1%)	25 (16.1%)	

C.I., confidence intervals. CHO, cholesterol. HDL, high-density lipoprotein. LDL, low-density lipoprotein. TG, triglyceride. VL, plasma HIV-RNA viral load. * Statistically significant, *p* value of <0.05. Group-1: naïve; group-2: HAART-treated.

**Table 4:** Metabolic syndrome among naïve and HAART patients by age (*n* = 199).

Range of Age	Group-1	MetS	%	Group-2	MetS	%	*p* Value	Total (G-1 + G-2)	MetS	%
20–30	24	2	8%	57	15	26%	0.0810	81	17	21%
31–40	15	5	33%	78	20	26%	0.5373	93	25	27%
41–50	3	1	33%	14	7	50%	1.0000	17	8	47%
>50	2	0	0%	6	6	100%	0.0357 *	8	6	75%
Total	44	8	18%	155	48	31%	0.1281	199	56	28%

Group-1: naïve; group-2: HAART-treated. MetS was defined as the presence of 3 or more of the following 5 abnormalities for men: (1) waist ≥ 90 cm, (2) systolic blood pressure (SBP) ≥ 131 mmHg or diastolic blood pressure (DBP) ≥ 81 mmHg, (3) HDL < 40 mg/dL, (4) fasting glucose ≥ 100 mg/dL, and (5) triglyceride (TG) ≥ 150 mg/dL. * Statistically significant, *p* value of <0.05 was estimated by Fisher’s exact test.

**Table 5:** Cardiovascular risk among naïve and HAART participants (*n* = 196).

Items	Total	Group-1 (±SD)	Group-2 (±SD)	*p* Value
Numbers	196	42	154	
FRS (%)	4.53	4.70 (±4.20)	3.87 (±5.92)	0.3956
Age (mean)	32.9	29.95 (±7.18)	33.70 (±8.32)	0.0084 *
Heart age/vascular age (mean)	38	36.00 (±12.14)	38.00 (±13.80)	0.3946

Group-1: naïve; group-2: HAART-treated. * Statistically significant, *p* value of <0.05 was estimated by Fisher’s exact test.

**Table 6:** Multiple comparison between every two HAART regimens based on lipid profiles by the Tukey honest significant difference (HSD) test.

Variable		(I) Drug	(J) Drug	Average Difference (I-J)	SE	Sig.	95% C.I.	
							Low	High
TG	Tukey HSD (TG)	PIs	NNRTIs	41.202 *	13.953	0.010 *	8.17	74.23
			InSTIs	52.636 *	21.118	0.036 *	2.64	102.63
		NNRTIs	PIs	−41.202 *	13.953	0.010 *	−74.23	−8.17
			InSTIs	11.434	20.772	0.846	−37.74	60.60
		InSTIs	PIs	−52.636 *	21.118	0.036 *	−102.63	−2.64
			NNRTIs	−11.434	20.772	0.846	−60.60	37.74
	Tukey HSD (CHO)	PIs	NNRTIs	5.361	6.001	0.645	−8.85	19.57
			InSTIs	16.834	9.083	0.156	−4.67	38.33
		NNRTIs	PIs	−5.361	6.001	0.645	−19.57	8.85
			InSTIs	11.473	8.933	0.406	−9.67	32.62
		InSTIs	PIs	−16.834	9.083	0.156	−38.33	4.67
			NNRTIs	−11.473	8.933	0.406	−32.62	9.67
CHO	Tukey HSD (HDL)	PIs	NNRTIs	−1.898	1.841	0.559	−6.26	2.46
			InSTIs	2.063	2.787	0.740	−4.53	8.66
		NNRTIs	PIs	1.898	1.841	0.559	−2.46	6.26
			InSTIs	3.961	2.741	0.321	−2.53	10.45
		InSTIs	PIs	−2.063	2.787	0.740	−8.66	4.53
			NNRTIs	−3.961	2.741	0.321	−10.45	2.53
	Tukey HSD (LDL)	PIs	NNRTIs	3.823	5.124	0.736	−8.31	15.95
			InSTIs	11.296	7.755	0.315	−7.06	29.65
		NNRTIs	PIs	−3.823	5.124	0.736	−15.95	8.31
			InSTIs	7.474	7.628	0.591	−10.58	25.53
		InSTIs	PIs	−11.296	7.755	0.315	−29.65	7.06
			NNRTIs	−7.474	7.628	0.591	−25.53	10.58

CHO, cholesterol. HDL, high-density lipoprotein. HSD, honest significant difference test. InSTIs, entry inhibitors and integrase strand transfer inhibitors. LDL, low-density lipoprotein. NNRTIs, nonnucleoside reverse transcriptase inhibitors. NRTIs, nucleoside reverse transcriptase inhibitors. PIs, protease inhibitors. TG, triglyceride. * Statistically significant, *p* value of <0.05.

## Data Availability

The datasets generated during and/or analyzed during the current study are available from the corresponding authors on reasonable request.

## Supplementary Materials

The following are available online at https://www.mdpi.com/article/10.3390/medicina57060578/s1, Supplemental Table S1. Cardiovascular risk among the naïve and HAART patients by age (*n* = 196); Table S2. Lipid profiles among the three HAART regimens; Table S3. ANOVA test of TG, CHO, HDL and LDL significance among the three HAART regimens.
