# Supplementary material for: Metabolic Syndrome Prevalence and Cardiovascular Risk Assessment in HIV-Positive Men with and without Antiretroviral Therapy
Source: Medicina (Kaunas). 2021 Jun 5;57(6):578. doi: 10.3390/medicina57060578 (PMC8230309; doi:10.3390/medicina57060578)
Supplement: Supplementary file 1 [file medicina-57-00578-s001.zip › medicina-1211107-supplementary.pdf]

Supplemental Table S1.

Cardiovascular risk among the naïve and HAART patients by age (N=196).

Range of age	Group-1	FRS % (±SD)	Group-2	FRS % (±SD)
20-30	24	1.65 (±1.11)	56	1.43 (±1.05)
31-40	15	5.32 (±3.46)	77	4.18 (±2.90)
41-50	3	14.45 (±4.50)	13	10.85 (±6.58)
>50	0	--	8	22.73 (±7.79)

Group-1: Naïve; Group-2: HAART

Supplemental Table S2. Lipid profiles among the three HAART regimens.

Descriptive statistics		N	mean (mg/dl)	SD	SE	95% C.I. Low	95% C.I. High	Mini (mg/dl)	Maxi (mg/dl)
TG	PIs	62	162.95	87.150	11.068	140.82	185.08	45	395
	NNRTIs	72	121.75	77.192	9.097	103.61	139.89	27	388
	InSTIs	19	110.32	69.343	15.908	76.89	143.74	37	354
	total	153	137.03	82.913	6.703	123.78	150.27	27	395
CHO	PIs	62	170.10	33.778	4.290	161.52	178.67	108	269
	NNRTIs	72	164.74	37.007	4.361	156.04	173.43	92	322
	InSTIs	19	153.26	27.000	6.194	140.25	166.28	106	196
	total	153	165.48	34.807	2.814	159.92	171.04	92	322
HDL	PIs	62	39.50	11.405	1.448	36.61	42.40	20	73
	NNRTIs	72	41.40	9.984	1.177	39.06	43.75	20	70
	InSTIs	19	37.44	10.352	2.375	32.45	42.43	22	59
	total	153	40.14	10.643	.860	38.44	41.84	20	73
LDL	PIs	62	100.82	31.805	4.039	92.75	108.90	36	194
	NNRTIs	72	97.00	29.506	3.477	90.07	103.93	24	219
	InSTIs	19	89.53	20.654	4.738	79.57	99.48	54	124
	total	153	97.62	29.592	2.392	92.89	102.35	24	219

SD: Standard deviation, SE: Standard error (SE), C.I.: Confidence interval

PIs: Lopinavir/Ritonavir (n=28); Darunavir (n=14); Atazanavir (n=20).

NNRTIs: Nevirapine (n=24); Efavirenz (n=10); Rilpivirine (n=30); Efavirenz/Tenofovir/Emtricitabine (n=8).

InSTIs: Raltegravir (n=19). Two patients have no HAART data.

Supplemental Table S3.

ANOVA test of TG, CHO, HDL and LDL significance among the three HAART regimens

ANOVA

		Sum of Squares	df	Mean Square	F	Sig.
TG	Between Groups	72029.435	2	36014.718	5.553	.005*
	Within Groups	972910.460	150	6486.070		
	Total	1044939.895	152			
CHO	Between Groups	4197.119	2	2098.560	1.749	.177
	Within Groups	179955.090	150	1199.701		
	Total	184152.209	152			
HDL	Between Groups	278.118	2	139.059	1.231	.295
	Within Groups	16940.614	150	112.937		
	Total	17218.732	152			
LDL	Between Groups	1908.228	2	954.114	1.091	.339
	Within Groups	131195.785	150	874.639		
	Total	133104.013	152			

* Statistically significant, p value of <0.05.
